# Continuous mobile measurement of camptocormia angle using four accelerometers

**DOI:** 10.1007/s11517-024-03149-1

**Published:** 2024-06-27

**Authors:** K. Naderi Beni, K. Knutzen, J. P. Kuhtz-Buschbeck, N. G. Margraf, R. Rieger

**Affiliations:** 1 Chair of Networked Electronic Systems, Kiel University, Kiel, Germany; 2 Neurological Clinic, UKSH, Kiel, Germany; 3 Institute of Physiology, UKSH, Kiel, Germany

**Keywords:** Parkinson’s disease, Camptocormia, Malleolus method, Perpendicular method, IMU, Accelerometer, Wearable, Home assessment

## Abstract

Camptocormia, a severe flexion deformity of the spine, presents challenges in monitoring its progression outside laboratory settings. This study introduces a customized method utilizing four inertial measurement unit (IMU) sensors for continuous recording of the camptocormia angle (CA), incorporating both the consensual malleolus and perpendicular assessment methods. The setup is wearable and mobile and allows measurements outside the laboratory environment. The practicality for measuring CA across various activities is evaluated for both the malleolus and perpendicular method in a mimicked Parkinson disease posture. Multiple activities are performed by a healthy volunteer. Measurements are compared against a camera-based reference system. Results show an overall root mean squared error (RMSE) of 4.13° for the malleolus method and 2.71° for the perpendicular method. Furthermore, patient-specific calibration during the standing still with forward lean activity significantly reduced the RMSE to 2.45° and 1.68° respectively. This study presents a novel approach to continuous CA monitoring outside the laboratory setting. The proposed system is suitable as a tool for monitoring the progression of camptocormia and for the first time implements the malleolus method with IMU. It holds promise for effectively monitoring camptocormia at home.

## Introduction

As the aging population grows worldwide, the prevalence of neurological disorders, including Parkinson’s disease (PD), is also increasing [1–3]. PD is the fastest-growing and the second most common neurodegenerative disorder in the elderly population, with a considerable impact on the healthcare system [4–6]. PD is a neurological disorder commonly associated with motor symptoms including tremor, bradykinesia (slowness of movement and speed), rigidity (stiffness and inflexibility of muscles), and akinesia (lack of movement) [7].

One of the major complications of advanced PD is postural abnormalities, mainly camptocormia [8]. Camptocormia is a pathological non-fixed forward flexion of the trunk that occurs involuntarily during standing, walking, and sitting. It affects about 7% of the patients with PD [9]. Regularly, it is the main complaint for these patients causing relevant back pain and reducing their mobility and autonomy resulting in a reduced quality of life [10].

**Fig. 1:**
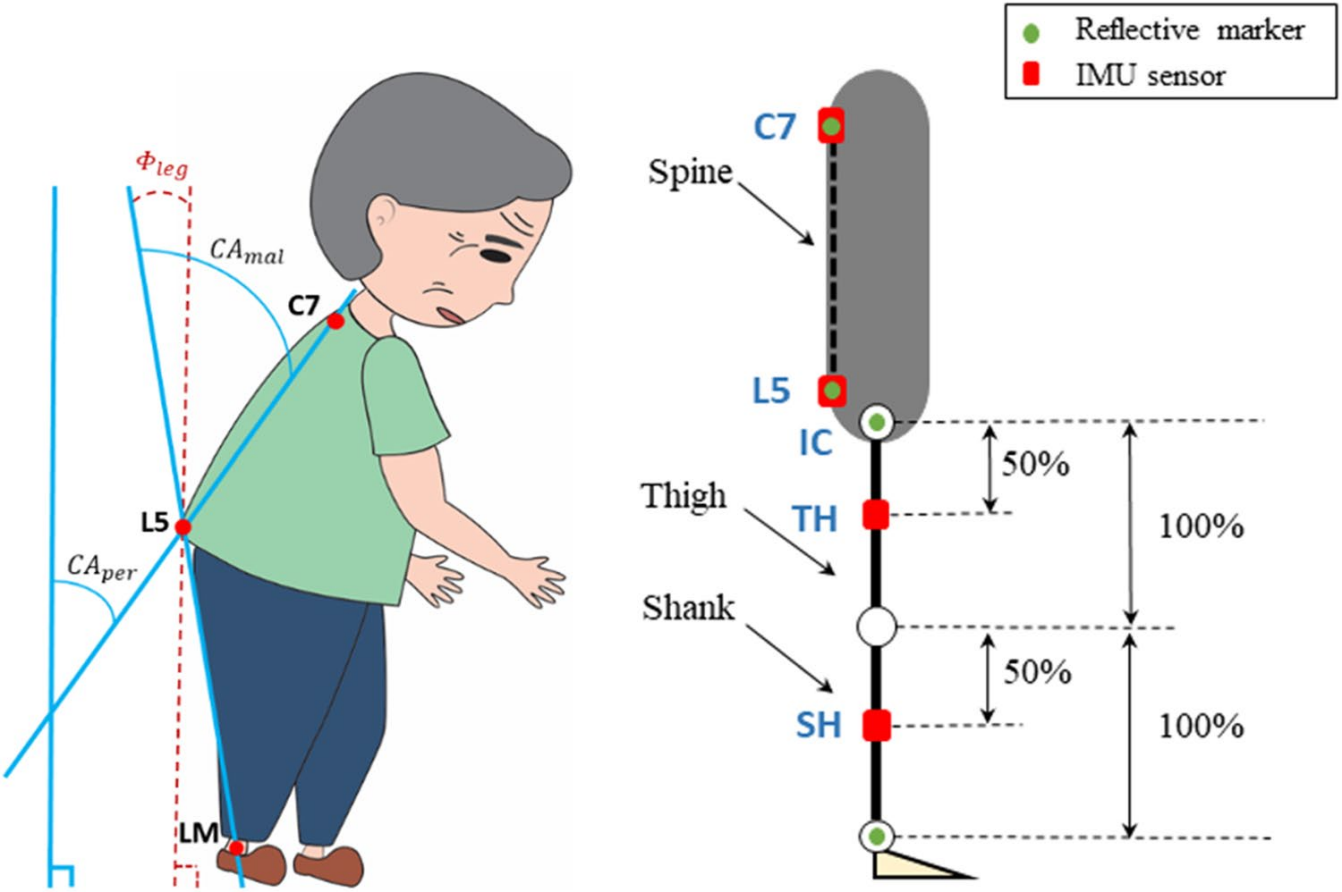
Spinous process of C7, L5, and LM is used for the measurement of the camptocormia angles, which are calculated with the perpendicular (CAper) and the malleolus (CAmal) methods (left). Location of sensors and reflective reference markers on the body (right)

As research in the field of camptocormia is progressing, it is increasingly important to define reliable and valid outcome parameters for treatment studies. A suitable candidate is the forward bending angle of the trunk as it is also one of the important defining aspects of the syndrome. In a recent paper [10], a cutoff criterion for camptocormia is defined as a forward flexion with an angle exceeding 30° when assessed by the malleolus method as the recommended method according to an international consensus paper [11] and the Movement Disorder Society (MDS) [12]. An alternative method that is also discussed is the perpendicular method that is still lacking an empirically validated cutoff criterion for the definition of camptocormia [11]. In the perpendicular method, the CA is defined as the angle between an imaginary line connecting vertebrae L5 to C7 and the vertical to ground as shown in Fig. 1. In the malleolus method, it is defined as the angle between a line connecting vertebrae L5 to C7 and a second line connecting vertebra L5 to the lateral malleolus (LM) of the foot. Generally, the CA measured by the perpendicular method is systematically lower than that measured by the malleolus method, and the perpendicular method is less sensitive to changes in clinical trials.

The CA can be manually measured using patients’ photographs or short sequences of videos, which limit the measurement to a specific location and time [11, 13, 14]. To date, precise estimation of the CA has been challenging due to the possible variability over the course of the day and based on clinical experience due to the susceptibility of measurements to the clinical setting and the attentiveness of patients being assessed. Other factors including placebo effects, the physical and mental state of the patient, or adaptation to the measurement environment can also have a significant impact on the assessment of therapy. Therefore, it is desirable to measure CA continuously over several hours and in the patient’s daily life to eliminate these factors [15].

The aim of this study is to propose a customized method for measuring the CA and to validate its accuracy with a healthy volunteer. The method uses four Inertial Measurement Unit (IMU) sensors and is able to implement both the perpendicular and the malleolus methods. To the best of our knowledge, this is the first study to investigate the feasibility of using IMU sensors to measure CA with the malleolus method. We measure the CA using both static (standing still upright and standing still with forward bending) and dynamic (walking, Parkinsonian gait) positions performed by the volunteer. It is insightful to investigate CA in dynamic conditions as it can potentially be affected by and during these activities.

The principle reliability of IMU for spine and joint kinematics and posture assessment has been shown in recent related works. The authors in [16] developed a cost-effective wearable system for real-time posture measurement and feedback, demonstrating its efficacy in improving posture and reducing pain. While promising, the study acknowledges the need for larger clinical trials and the limited efficacy in severe spinal conditions. In [17] the between-day reliability of IMUs in measuring spine movement quality in chronic low back pain patients was investigated, highlighting the potential for aiding diagnosis and treatment in clinical settings. Work [18] introduced a wireless sensor system for quantifying spine posture and movement during daily activities, emphasizing its role in remote monitoring of lower back pain. In [19] a non-invasive approach for accurate spine posture assessment using inertial or optical sensors was proposed, with potential applications in clinical settings for the evaluation and monitoring of spinal disorders. Study [20] advanced the field by developing a deep learning model for predicting joint moments and ground reaction forces using IMU sensors, with implications for clinical analysis and assistive device control. In [21] a validation study compared IMU systems with optoelectronic systems, highlighting the accuracy and reliability of wearable systems for clinical gait analysis. The IMU-based system presented in [22] for real-time measurement during total hip replacement surgeries offered a radiation-free alternative to CT scans for precise angle measurement. Finally, [23] proposed a fusion of smartphone cameras and IMUs for estimating biomechanical outcomes, showcasing its potential as a portable gait assessment tool for various applications including outcome assessment after knee surgery and gait training. This synthesis of research highlights the diverse methodologies, findings, and potential applications of wearable sensor technologies in assessing spine movements and related clinical interventions. Table 1 summarizes the key points of these works.

**Table 1:** Summary of recent studies in wearable sensor technology for spine and joint kinematics assessment

Study	Methodology	Key findings	Main contribution	Limitations	Potential application
Rodriguez et al. [16]	Utilized IMUs with accelerometers, gyroscopes, and magnetometers. Fuzzy system controlled vibration unit for posture correction.	Validation: rms deviation ≤1.24°. Pilot study: improved posture and reduced pain. Estimated cost: < U.S. $100.	Developed cost-effective wearable for real-time posture measurement and feedback. Validation in lab and clinic.	Need for larger clinical trials. Limited efficacy in severe spinal conditions. Cost estimate based on assumptions.	Suitable for postural therapy, posture awareness enhancement, and effective physical therapy in clinical and home settings.
B. Graham et al. [17]	Assessed between-day reliability of HIKOB FOX IMU in measuring local dynamic stability (LDS) and variability of trunk movements in chronic low back pain (LBP) patients	Average coefficient of variation (CV) for *λ*max: ~10% in flexion/extension and rotation tasks, <20% including complex task. - Intraclass correlation coefficient (ICC) for *λ*max ranged from 0.28 to 0.81. - Reliability better for *λ*max than MeanSD. - Moderate reliability of *λ*max in sagittal and transverse planes, worse for lumbar spine.	Demonstrated moderate between-day reliability of IMU in assessing spine movement quality in LBP patients.	Limited sample size; reliability not assessed in specific LBP subgroups.	Assessment of spine movement quality and variability in clinical settings, aiding diagnosis and treatment of LBP patients.
Moon et al. [18]	Developed a fully wireless multi-sensor cluster system to monitor spine movements. Used custom-designed robotic lumbar spine simulator to generate reference sensor data. Employed mechanical motion templates for automated sensor pattern recognition for diagnosing LBP.	Lumbopelvic movements identified as significant risk factors for LBP. - Speed, angle range, acceleration, and intensity were used to characterize movements in individuals with and without LBP. - Need for an unobtrusive small wireless sensor system to quantify spine posture and movement in everyday activities.	Introducing a novel approach for detecting specific lumbopelvic movements with a wireless network system.	Lower accuracy of IMU-based motion measurement compared to optical camera-based methods. Small sample size for human subject testing.	Remote monitoring of LBP using a wearable sensor system to provide essential information for users and healthcare communities.
Michaud et al. [19]	Developed a procedure using inertial or optical sensors to construct subject-specific spine models, estimating positions and orientations of the 17 vertebrae.	Mean position error < 12 mm, validating method effectiveness. - Provides non-invasive tools for accurate spine posture assessment, overcoming traditional imaging limitations.	Introduces a novel approach for non-invasive spine posture determination, addressing calibration, scaling, and gender differences.	Limited validation on healthy subjects; further validation is needed against X-ray imaging for spinal deformities.	Clinical assessment of spine posture for prevention, evaluation, treatment, and monitoring of spinal disorders.
Hossain et al. [20]	Developed Kinetics-FM-DLR-Ensemble-Net, a deep learning model using three IMU sensors on the thigh, shank, and foot for joint moments and GRF prediction.	The first study to implement joint moments and GRF estimation in multiple walking conditions using IMU sensors via deep learning. - Outperforms state-of-the-art models for kinetics estimation.	Introduced a deep learning model for accurate estimation of joint moments and GRFs in diverse walking conditions, advancing existing methodologies.	A relatively large number of IMU sensors were used. Limited training data for specific walking conditions. The bagging technique increases computational complexity.	Clinical analysis, assistive device control, and kinetics measurement outside the lab.
Saggio et al. [21]	Validation study comparing an inertial-sensor-based system (Movit System G1) with an optoelectronic gold standard system (Vicon)	Very good measurement accuracy of the inertial-based system for hip, knee, and ankle ROMs in the sagittal plane during walking (RMSE < 2.66°, PCC > 0.97) - Good agreement between the two systems for spatio-temporal parameters (percentage errors <5%)	Demonstrated the accuracy and reliability of the inertial-sensor-based system compared to the optoelectronic gold standard system - Highlighted the potential of wearable systems as alternatives to traditional optoelectronic systems	Limitations in measuring knee and ankle movements in the frontal plane - Moderate correlation between systems in the transversal plane - Slight differences in ROM measurements between systems	Clinical gait analysis - Assessment of motor abilities in patients with neurological and orthopedic disorders - Evaluation of therapy efficacy and progress
Chen et al. [22]	IMU-based system for real-time measurement of pelvis and prosthesis angles during THR surgeries.	Accurate real-time measurement of implant angles with RMSE < 3.52° under fixed angles and < 3.27° under changing angles. Real-time monitoring of prosthesis dislocation.	Optimization of quaternion-based algorithm for precise angle measurement. Development of wireless, reliable IMU system. Design of real-time protractor device.	Potential drift in IMU sensors. Errors in reference values and experimental setup.	Radiation-free alternative to CT scans for precise angle measurement during THR surgeries. Enhanced surgical precision and reduced complications. Clinical application in THR surgeries for improved outcomes.
Tan et al. [23]	Fusion of smartphone cameras and IMUs. Development of deep learning model.	Improved accuracy in estimating knee adduction moment (KAM) and knee flexion moment (KFM). RMSE: 0.49% BW⋅BH for KAM, 0.66% BW⋅BH for KFM.	Advancement in estimating biomechanical outcomes using smartphone cameras and IMUs.	Limited diversity in subjects. Model may require fine-tuning for broader populations.	Portable gait assessment tools for clinics, homes, and athletic facilities. Outcome assessment for knee surgery and gait training.

The remainder of the paper is organized as follows. Section 2 provides more detail about the measurement methods, the system setup, and data processing, and is followed by measured results in Section 3. A discussion is provided in Section 3.1 and conclusions in Section 5.

## Materials and methods

### Experiment setup

In the proposed configuration shown in Fig. 1, four identical IMU sensors (Physilog 6s, GaitUp, Switzerland) are used to measure the CA. The placement of sensors and reflective markers for a camera system, which was used as the reference, is also shown. Two of the sensors are affixed to the skin using medical tape near the fifth lumbar vertebra (L5) and seventh cervical vertebra (C7) respectively, while the other two are positioned on the middle of the thigh (TH) and shank (SH) respectively. Four reflective markers are used for reference, placed on C7 and L5 directly on the sensors, as well as markers directly on the right iliac crest (IC) and right lateral malleolus (LM) bone. Consistent with conventional static CA measurement, C7 is chosen since it has relatively low mobility compared to the other cervical vertebrae [24]. The placement of the IMU sensors on the leg was determined based on the work of Saito et al. [25]. Specifically, the IMU sensor on the thigh was positioned midway between the greater trochanter and knee joint, while the IMU sensor on the shank was located at 50% of the distance between the knee joint and the lateral malleolus (LM). The CA calculated with the new IMU sensor method does not refer to the isolated sagittal plane, but describes inclinations relative to the vertical, i.e., not only a forward tilt, but also lateral and oblique inclinations.

To conduct the study, a healthy 23-year-old female volunteer is recruited. The CA is measured during various activities including standing still, standing still with forward bending, walking, and mimicking Parkinsonian gait. All forward bending is performed predominately in the sagittal plane. Additional sideward bending, as may be the case in patients with additional Pisa syndrome, is not included here to obtain CA comparable with other methods. Accelerometer data are collected from the IMU sensors, which records in three spatial dimensions each. As four sensors are used, a total of 12 raw data channels are obtained over a recording period of 4 min (of which about 60, 50, 25, and 80 s include standing still, standing still with forward bending, walking, and mimicked Parkinsonian gait, respectively). The CA is then calculated from the recorded acceleration data after recording is completed. To evaluate the accuracy of the measurement, a commercial motion capture system (Miqus M3 Camera, Qualisys, Sweden) available in the kinematics laboratory of the Department of Neurology, University Hospital Schleswig-Holstein, Kiel, is employed as a reference. All experimental procedures involving human subjects were approved by the institution’s ethical review board and were conducted according to the Declaration of Helsinki.

### Computer-based data processing

After completion of the recording session, the raw data from all IMUs is uploaded to a PC using the data transfer interface provided by the manufacturer of the IMU sensors. This database is then processed using Matlab software (MathWorks, USA) as follows. A third-order Savitzky-Golay filter, which is a signal processing digital filter that fits a polynomial to a sliding window of neighboring data points, is applied to smooth the raw data that were sampled at 128 Hz. This filter is particularly effective for smoothing noisy data with minimal distortion of important features [26]. The filter order was empirically chosen as the results showed that it results in low deviation from the reference angle captured by the camera system.

**Fig. 2:**
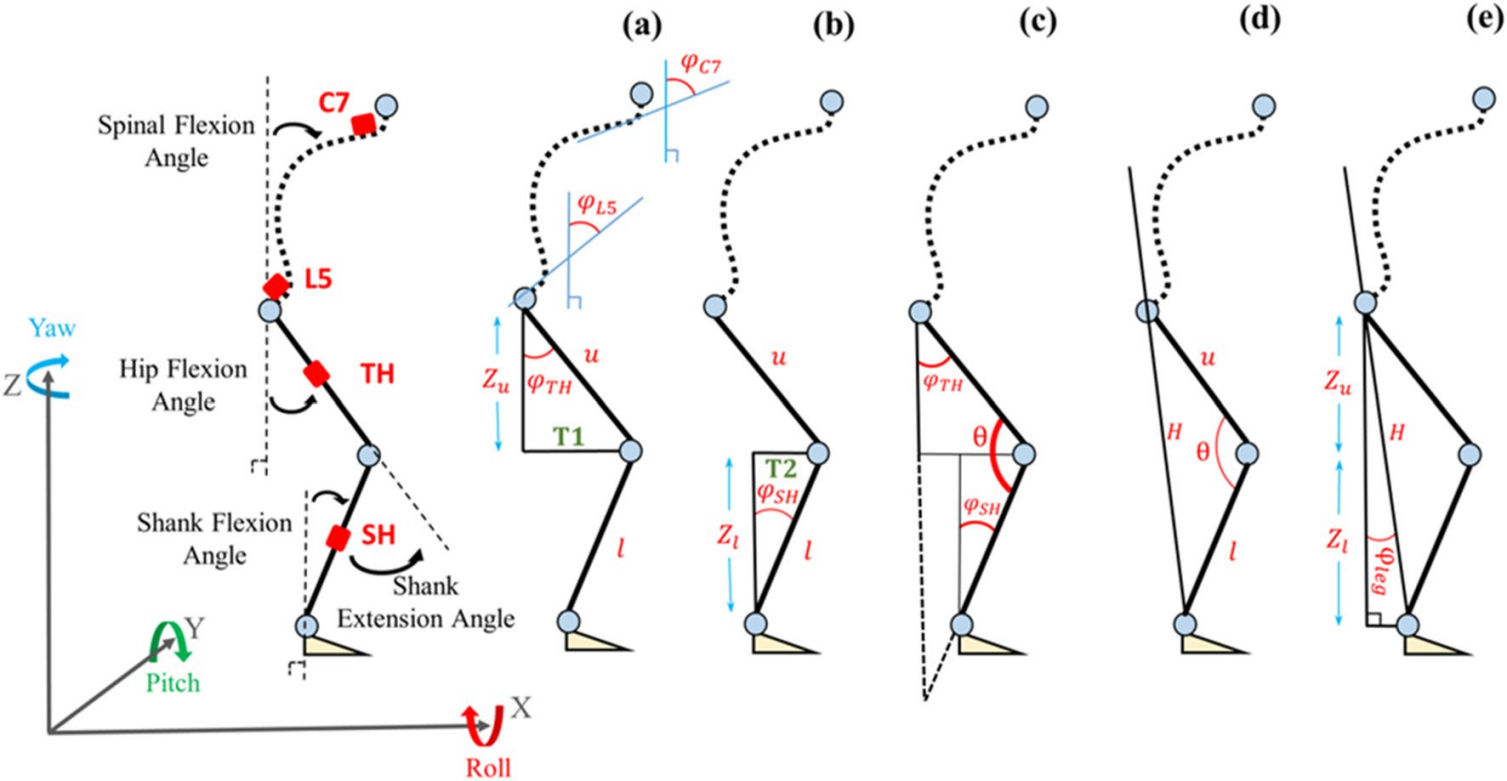
Schematic drawing illustrating spinal flexion, lower limb joint angles, sensor positions, and definitions used in the algorithm to calculate the leg angle

Obtaining the angle *CA*_*mal*_ according to the malleolus method involves two steps: First, the perpendicular angle *CA*_*per*_ between the lines of C7 to L5 and the vertical is calculated, and second the angle *Φ*_*leg*_ between the line of L5 to LM and the perpendicular is calculated (as shown in Fig. 1 on the left). In the following, lowercase symbols denote the angles measured by the sensors, while uppercase represents further processed angles such as leg and camptocormia angles. The algorithm and the underlying assumptions for measuring *CA*_*per*_ from the IMU sensor data C7 and L5 are discussed in the authors’ previous publication [27]. In that model, the spine is considered a flexible chain of vertebra with a defined height and mobility. Based on this assumption and the measured angle of gravity from the IMU sensors on L5 (*φ*_*L5*_) and C7 (*φ*_*C7*_), following model estimates *CA*_*per*_:

1 $$CA_{per} \left( {\phi_{L5} ,\phi_{C7} } \right) = 0.3856 + 0.4542\phi_{L5} + 0.5458\phi_{C7}$$

where all angles are expressed in degree. The inclinations *φ*_*C7*_ and *φ*_*L5*_ are calculated from accelerometer data [28]:

2 $$\phi_{C7,L5} = - \arctan \left( {\frac{{\sqrt {a_{y}^{2} + a_{z}^{2} } }}{{a_{x} }}} \right)$$

where *α*_*x*_,* α*_*y*_, and* α*_*z*_ are acceleration components measured in the three spatial directions defined in Fig. 2. Measurement with the malleolus method requires additional sensor information from the lower body. Since the leg bends at the knee joint, sensors on both the upper and lower legs are needed as a minimum. The human leg is conventionally represented as a system of seven degrees of freedom [29], but its complex nature makes it difficult to exactly replicate its joint kinematics. As a simplification, this study approximates leg movement as two coupled pendula rotating purely on the Y-axis. Fig. 2 shows the sensor placements in a typical standing position with slightly flexed trunk and legs. The leg position is retrieved from sensors TH and SH which yield the hip joint angle (*φ*_*TH*_) and the shank joint angle (*φ*_*SH*_) respectively:

3 $$\phi_{TH,SH} = - \arctan \left( {\frac{{a_{y} }}{{\sqrt {a_{x}^{2} + a_{z}^{2} } }}} \right)$$

From these data, *Φ*_*leg*_ is calculated with reference to the intermediate variables shown in Fig. 2. The length *u* of the upper leg and *l* of the lower leg are measured at the patient during sensor placement. *Z*_*u*_ and *Z*_*l*_ are thus calculated using the Pythagorean theorem (subfigures a and b). The knee angle *θ* = 180°-*φ*_*TH*_-*φ*_*SH*_ is obtained by considering the interior angles of triangles *T1* and *T2*, each summing to 180° (c). Based on the cosine law, length *H* is calculated using *u*, *l*, and *θ* (d). Finally, *Φ*_*leg*_ is obtained using the inverse cosine (e):

4 $$\Phi_{leg} = \arccos \left( {\frac{{Z_{u} + Z_{l} }}{H}} \right)$$

with $H = \sqrt {u^{2} + l^{2} - \left( {2 \cdot u \cdot l \cdot \cos \theta } \right)}$. Finally, the malleolus CA results as

5 $$CA_{mal} = CA_{per} + \Phi_{leg} .$$

**Fig. 3:**
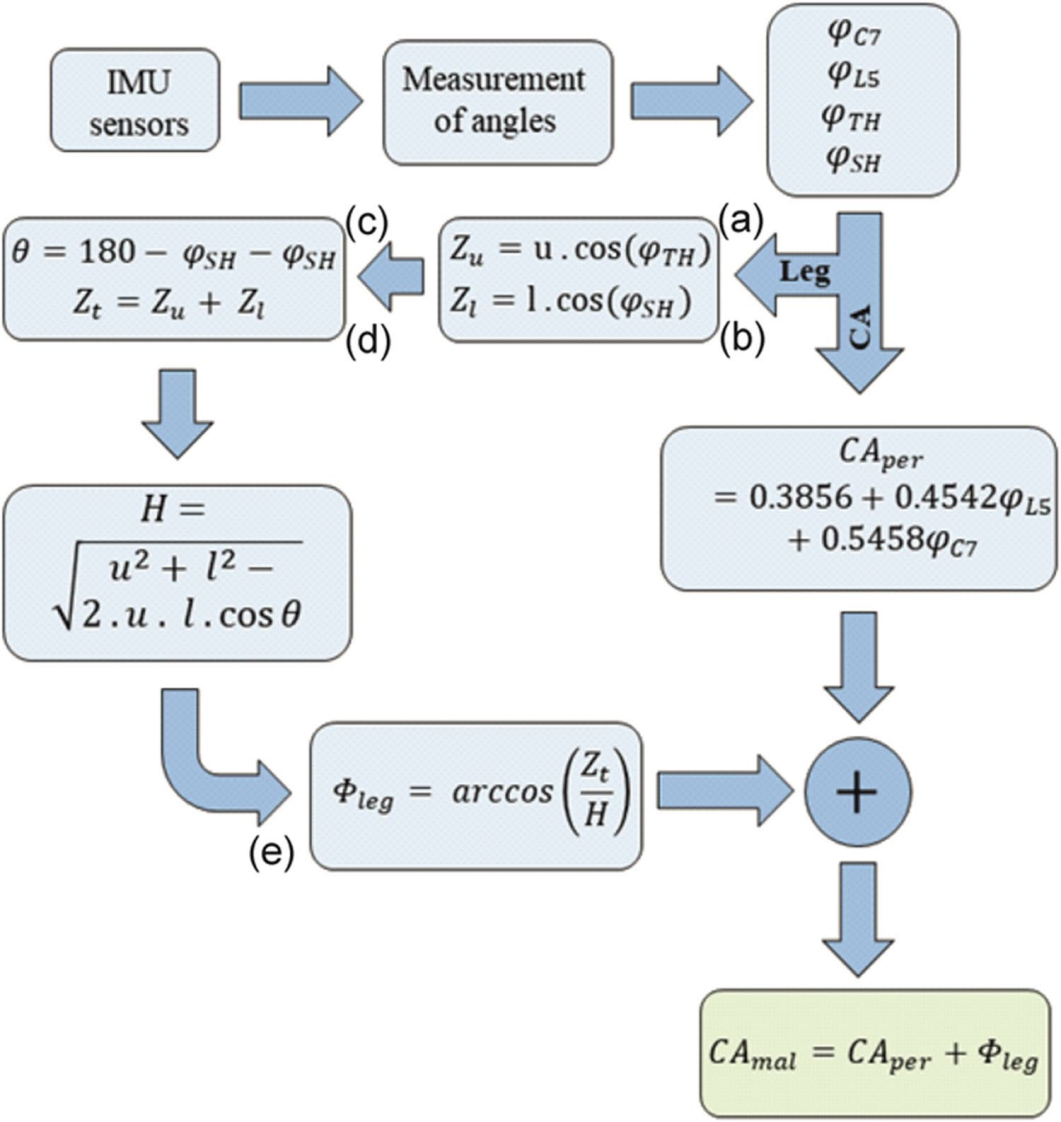
The data flow diagram of the system to measure CA with the malleolus method. Letters a-e in brackets refer to Fig. 2

This algorithmic flow is implemented in a Matlab post-processing routine and is summarized in Fig. 3.

CA_*per*_ and *Φ*_*leg*_ describe the inclinations of the trunk or, respectively, the leg relative to the vertical. CA_*per*_ does therefore capture forward, lateral, and oblique inclinations of the trunk. *Φ*_*leg*_ is positive when the foot is in front of the frontal plane (as in. Fig. 2e); otherwise, it is negative. CA_*mal*_ is the sum of both spatial angles. These spatial angles are not subject to distortions due to projection errors, which affect the CA when projected onto the sagittal plane (like on photos taken from the side), especially if forward flexion of the trunk is combined with a substantial lateral tilt or if the camera is unfavorably rotated.

**Fig. 4:**
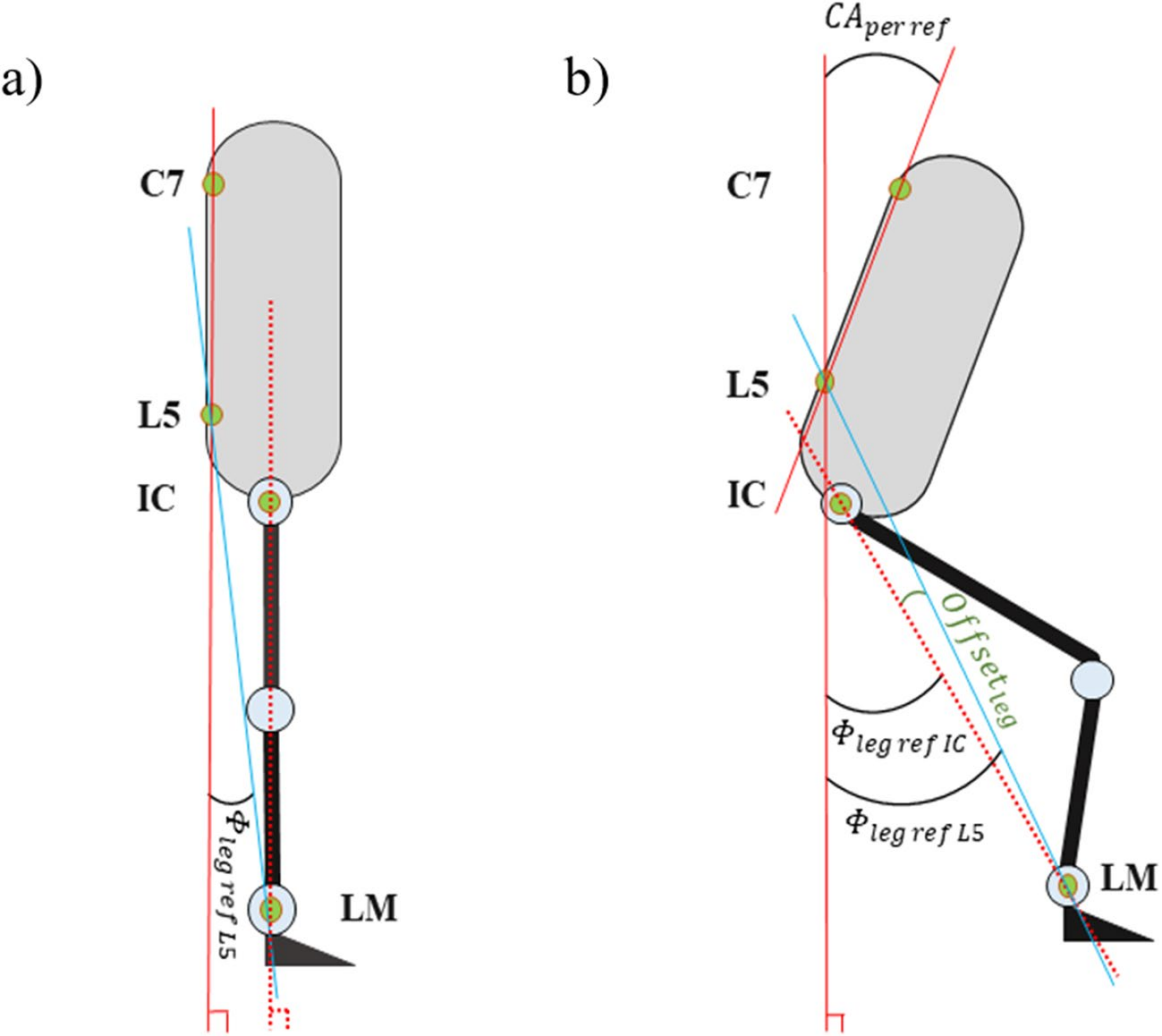
Using L5 as the reference point for leg angle measurement instead of the rotation point at IC yields a small measurement offset

In conventional kinematic algorithms, the reference point of a body segment is ideally located at the center of joints [30]. Here, however, we follow the established definition of the CA using L5 as the reference position, which does not coincide with the rotational axis of the hip joint located near the IC. Since the IMU located on the leg can only measure the leg angle with respect to the perpendicular, a small difference between the measured leg angle and the angle required for calculation of *CA*_*mal*_ exists. For example, Fig. 4a shows the case of a perfectly upright body where the angle of the leg towards the vertical is zero. Still, *CA*_*mal*_ is larger than zero since IC is located anterior to L5. For the relatively small range of angles involved even during motion (e.g., in Fig. 4b), the deviation is assumed to be practically constant (which is validated by measurements presented in Section 3), and we call this difference angle *offset*_*leg*_. Determining this offset once at the beginning of a measurement allows to subtract it from the recorded leg angle to obtain a more accurate *CA*_*mal*_.

The validity of the calculated angles is assessed by comparison with corresponding values reconstructed from the optical 3D motion capture data. These reference angles are obtained from the reflective markers. Using L5 as the reference marker for the leg angle, the angles are given as

6 $$CA_{ref\;L5} = \Phi_{per\;ref} + \Phi_{leg\;ref\;L5}$$

where

7 $$\Phi_{per\;ref} = \arctan \left( {\frac{{\sqrt {\left( {C7_{x} + L5_{x} } \right)^{2} + \left( {C7_{y} + L5_{y} } \right)^{2} } }}{{C7_{z} - L5_{z} }}} \right)$$

and

8 $$\Phi_{leg\;ref\;L5} = \left| {\arctan \left( {\frac{{\sqrt {\left( {LM_{x} + L5_{x} } \right)^{2} + \left( {LM_{y} + L5_{y} } \right)^{2} } }}{{L5_{z} - LM_{z} }}} \right)} \right|.$$

When IC is used as the reference angle (for comparisons in Section 2.1), *Φ*_*leg ref L5*_ is replaced by *Φ*_*leg ref IC*_:

9 $$\Phi_{leg\;ref\;IC} = \left| {\arctan \left( {\frac{{\sqrt {\left( {LM_{x} + IC_{x} } \right)^{2} + \left( {IC_{y} + LM_{y} } \right)^{2} } }}{{IC_{z} - LM_{z} }}} \right)} \right|.$$

#### Calibration methods for spinal process and lower limb angle measurements

Two different calibration procedures were considered during post-processing.

##### Zero calibration method

Calibration involves the continuous measurement of *φ*_*C7*_ and *φ*_*L5*_, *φ*_*TH*_, and *φ*_*SH*_ angles while the volunteer is standing still and upright for 60 s at the beginning of the recording. The average angle obtained during the 60-s period is used as the zero reference angle for the subsequent measurement. This method works well for healthy volunteers who are able to stand upright.

##### Patient-specific calibration

A digital photograph of the subject is taken in still standing position with forward lean at the beginning of the recording. It is uploaded into the *NeuroPostureApp* designed for manually measuring angles from images [11]. The app is a web-based tool that allows to manually mark the reference points on the image and that then calculates the ensuing CA from this input. This has been done with the perpendicular method as well as with the malleolus method and compared with the CA obtained from the IMU. The differences *offset*_*per*_ and *offset*_*mal*_ are subtracted from the subsequently recorded data. Since calibration takes place in the standing forward lean position, this position is expected to later yield the most accurate CA measurement. We expect that it is sufficient to identify one or two main activities for CA progression measurement and that an exact tracking during other activities is less important. Different activities could be selected and calibrated for in principle. However, we do not wish to impose specific postures on a patient for calibration. A natural and suitable position would be chosen as the calibration point, usually expected to be the standing with forward lean position used here.

Sources of potential error that remain and that are included in the evaluation data are measurement plane misalignment, inaccuracy of the assumptions regarding the bending radius of the spine on which Eq. (1) is based, remaining static sensor misalignments, dynamic misalignments, nonlinear portions of the L5 offset (Fig. 4).

## Measurement and results

**Fig. 5:**
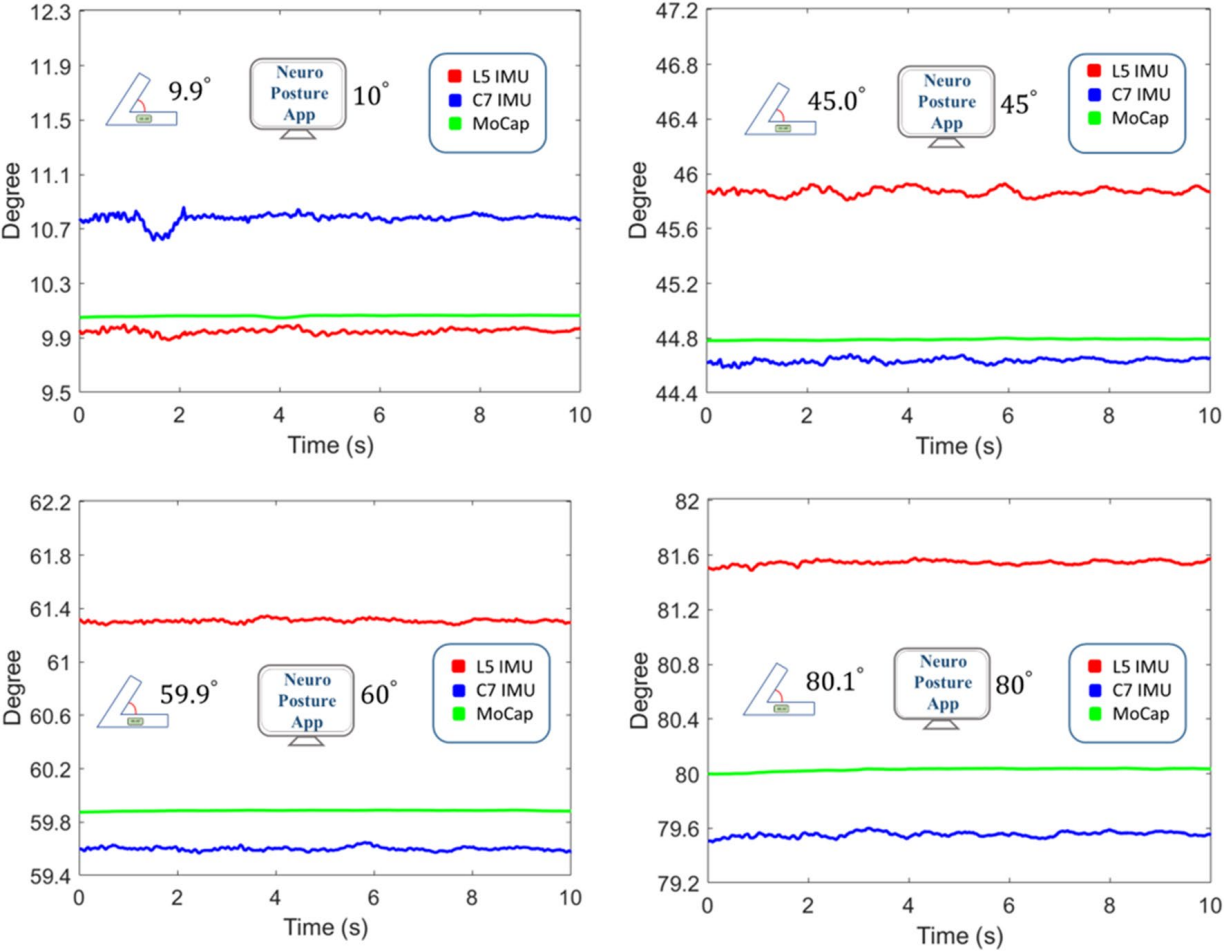
Comparison of tilt angles measured using the digital protractor, *NeuroPostureApp*, motion capture system, and two IMU sensors in a dummy test with a protractor

First, the matching of readings from the IMU sensors is verified by comparing with results obtained from the *NeuroPostureApp* and the motion capture system. The app is included into the comparison test, as it is an established tool used in previous studies, among others in [11, 31–33]. As we aim to maintain the link to results obtained in past studies, we demonstrate here that also compared to the app our IMU system yields significant results. Also, a first dummy test is performed using a digital protractor (*50440, BGS Technic Co, Germany*) with sensors and marker points attached. This test uses the exact IMUs placed at C7 and L5 during the in vivo recording. Employing the protractor allows to verify the CA algorithm in a static and well-controlled system, excluding any deviation due to motion. Due to its angled shape, the protractor provides a convenient means for setting defined angles between the attached sensors and thus serves as a stable reference base against which the correct operation of the other methods is tested. Reflective markers are attached on the sensors mounted on the head and end of the digital protractor slide. Angles are measured in 14 steps from 0° to 90° by adjusting the protractor opening angle. Still photos of the protractor are taken as basis for using the *NeuroPostureApp*. Fig. 5 shows results for four exemplary protractor angles. Overall, the four tools yield matching results with a difference of less than 2°.

**Fig. 6:**
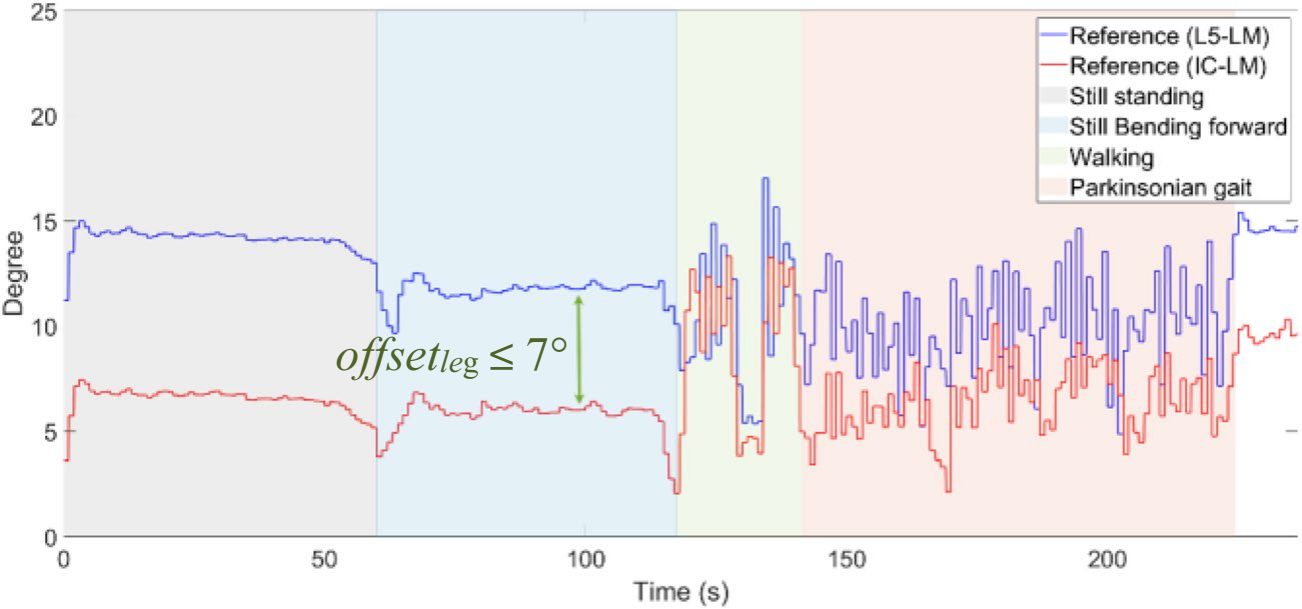
A section of reference leg angles obtained from the motion capture system. Angle *offset*_*leg*_ is the difference between the L5-LM reference (*Φ*_*leg ref*_ _*L5*_), and the IC-LM reference (*Φ*_*leg ref IC*_)

Second, the assumption of a small and approximately constant offset angle *offset*_*leg*_ is verified using a video recording from the camera system. The leg angles using L5-LM as well as IC-LM as their reference points are extracted from the video. Fig. 6 shows a section of the recording period with different performed activities. The maximum angular offset is estimated as about 7° within the section covering the standing still position with forward lean. For comparison, *offset*_*leg*_ is also estimated from a series of still photographs of 39 Parkinsonian patients with camptocormia taken from a former study [11]. The angles are manually measured using the *NeuroPostureApp*. The results yield an offset ranging from 4 to 9°, with an average value of 6.73°, which is close to the value obtained here from the video.

**Fig. 7:**
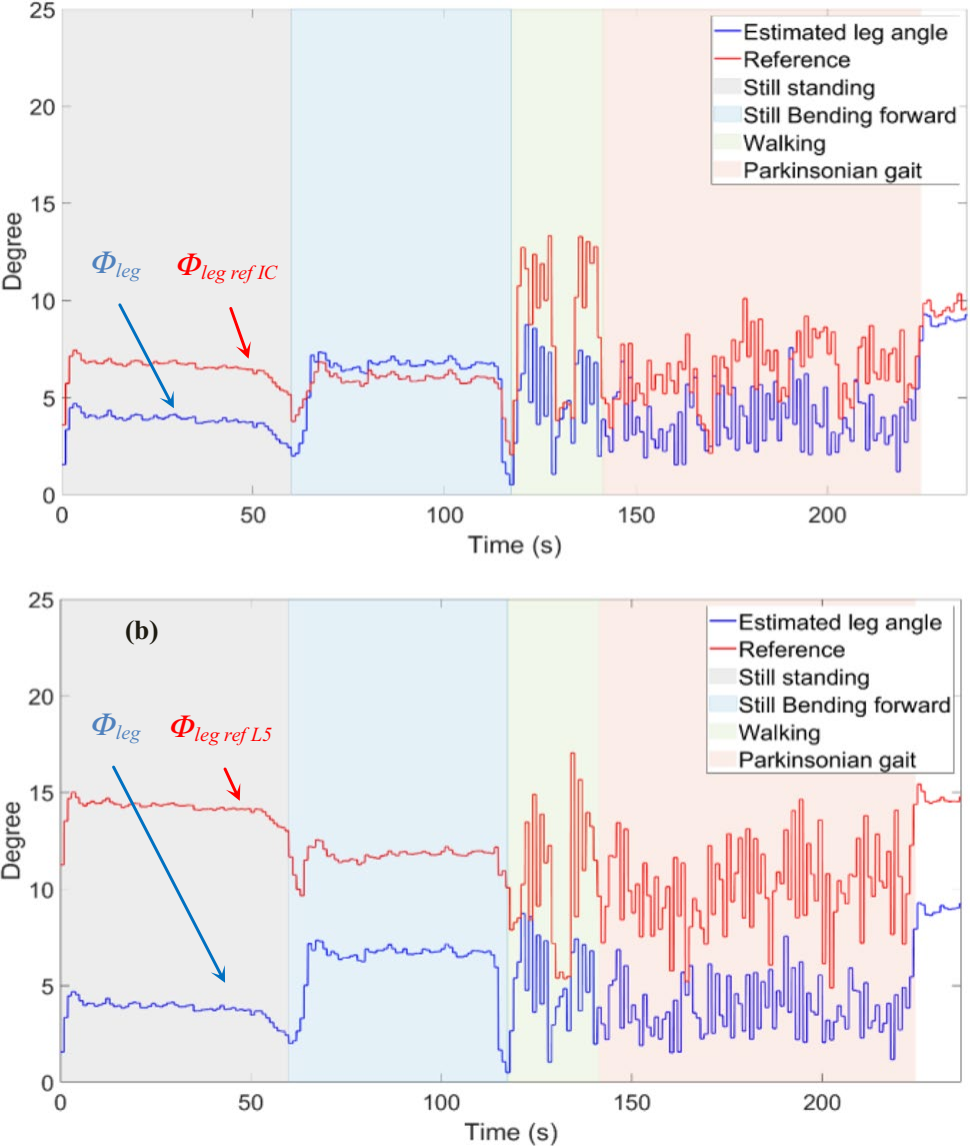
A section of leg angle values obtained from recorded IMU data using (4) in comparison to the reference angle from the motion capture system, using reference frame of IC and LM (**a**) and L5 and LM (**b**). The healthy volunteer was standing still, flexing her trunk, walking, and mimicking the parkinsonian gait as indicated

Third, the results of the proposed measurement setup and processing algorithm for the malleolus method are compared with measurements obtained from the camera reference. Fig. 7 shows the difference between* Φ*_*leg*_ and *Φ*_*leg ref L5*_, as well as *Φ*_*leg ref IC*_ respectively, after applying the zero calibration method. The angles were averaged over one second time windows. As expected, *Φ*_*leg ref IC*_ is close to *Φ*_*leg*_, whereas *Φ*_*leg ref L5*_ appears shifted by an offset angle of approximately 7°. It is also observed that the movement of the pelvis affects the leg angle as L5 rotates around IC. Table 2 presents the root mean squared error (RMSE) between *Φ*_*leg*_ and the offset corrected reference angles for different activities. The RMSE ranges from 0.91 to 4.69° for IC as reference and 1.68 to 4.16° for L5 as reference. It confirms that the correction with a constant offset yields useful results and that the IMU measurement tracks well with the camera reference. The overall RMSE over all activities is 2.72° and 2.62° for the IC–LM and L5-LM reference frames, respectively.

**Table 2:** Comparison of RMSE of leg angle with the reference frame L5-LM and IC-LM

Reference frame	Standing still upright		Standing still with forward lean	Walking	Parkinsonian gait	All activities
IC-LM (*Φ*_*leg*_− *Φ*_*leg ref IC*_)	2.74°		0.91°	4.69°	2.94°	2.72°
L5-LM (*Φ*_*leg*_−* Φ*_*leg ref IC*_ + *offset*_*leg*_)	3.63°		1.72°	4.16°	1.68°	2.62°

**Fig. 8:**
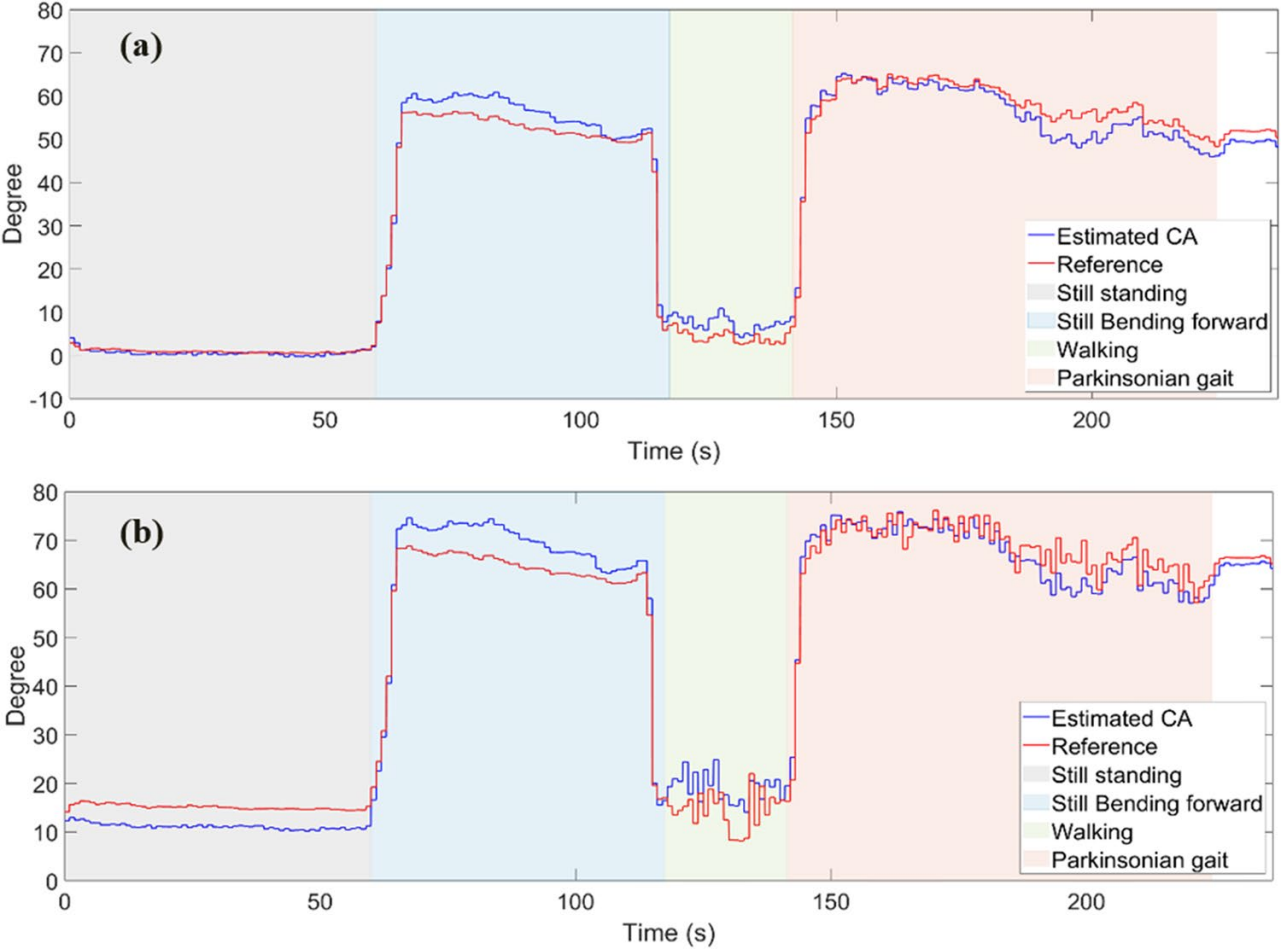
A section of CA values with perpendicular method added to *offset*_*per*_ (**a**), and CA values with malleolus method added to *offset*_*mal*_ (**b**). Offsets are obtained from the recorded IMU data using (1) and (4) in comparison to the reference angle from the motion capture system. L5-LM is used as frame for the leg angle portion. The healthy volunteer was standing still, flexing her trunk, walking, and mimicking Parkinsonian gait as indicated

**Fig. 9:**
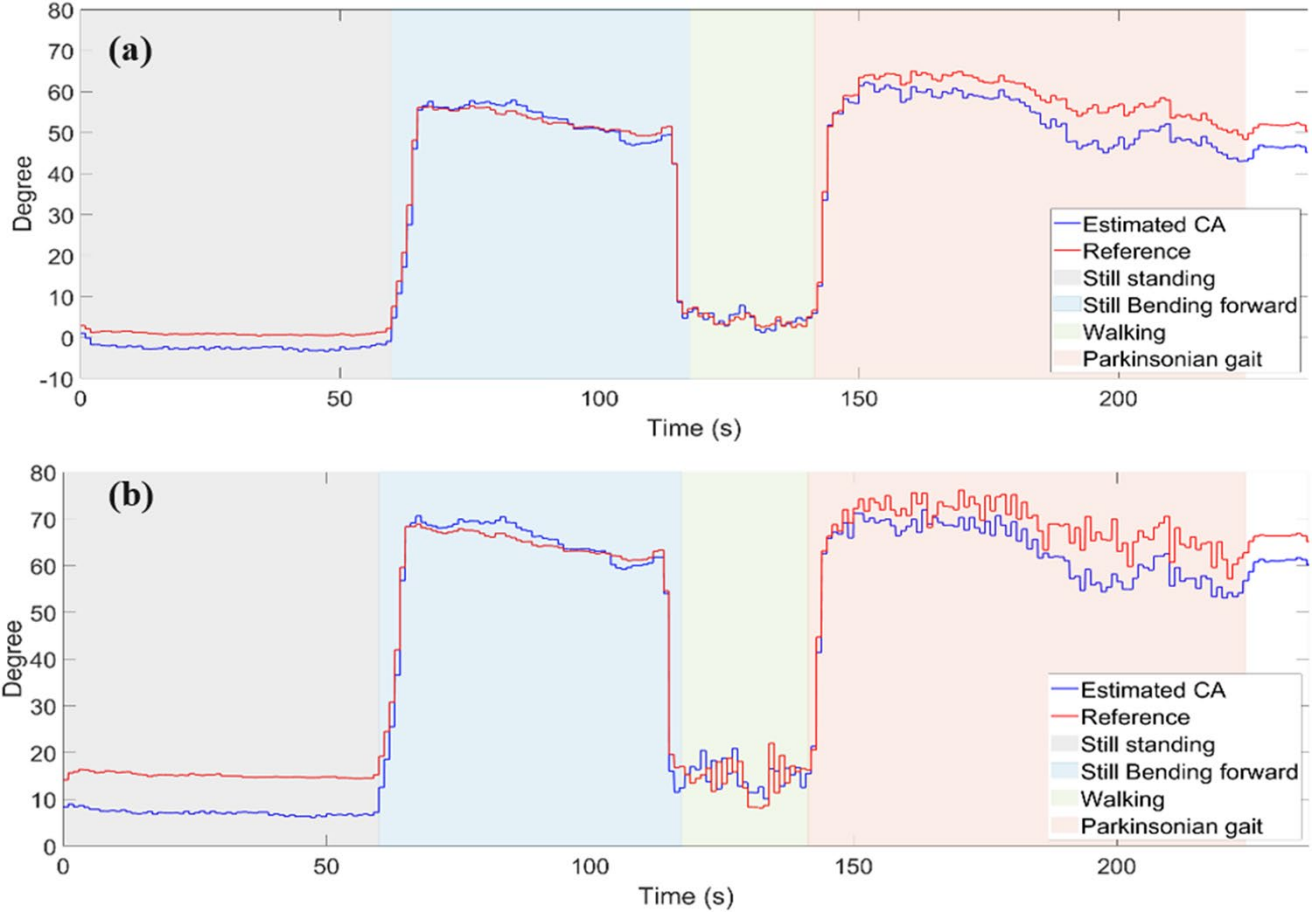
A section of CA values with perpendicular (**a**), and malleolus (**b**) methods obtained from the recorded IMU data using (1) and (4) in comparison to the reference angle from the motion capture system. L5-LM is used as frame for the leg angle portion. The healthy volunteer was standing still, flexing her trunk, walking, and mimicking Parkinsonian gait as indicated

Fourth, the differences using the perpendicular versus the malleolus method are examined. Fig. 8 shows the results for CA for the different activities using the L5-LM frame for the CA with zero calibration method and in Fig. 9 after patient-specific calibration. Table 3 presents RMSE values for both methods and for both calibration methods. The perpendicular method with applied zero calibration method shows the lowest average error for all activities, ranging from 0.53 to 3.32°. The malleolus method with zero calibration method shows the highest RMSE, ranging from 3.23 to 6.04°. The patient-specific calibration decreases the RMSE in the standing still position with forward lean to 1.68° and 2.45° for the perpendicular and malleolus methods, respectively (Fig. 9).

**Table 3:** Comparison of RMSE for the estimated CA and reference angles using the malleolus and the perpendicular method for several activities. L5-LM frame is used for the leg angle portion of CA

Calibration	Method	Standing still upright	Standing still with forward lean	Walking	Parkinsonian gait	All activities
Zero calibration method	Perpendicular	0.53°	3.31°	3.32°	3.02°	2.71°
	Malleolus	4.01°	4.79°	6.04°	3.23°	4.13°
Patient-specific calibration	Perpendicular	3.37°	1.68°	1.15°	5.5°	3.98°
	Malleolus	7.99°	2.45°	4.09°	6.21°	5.86°

**Fig. 10:**
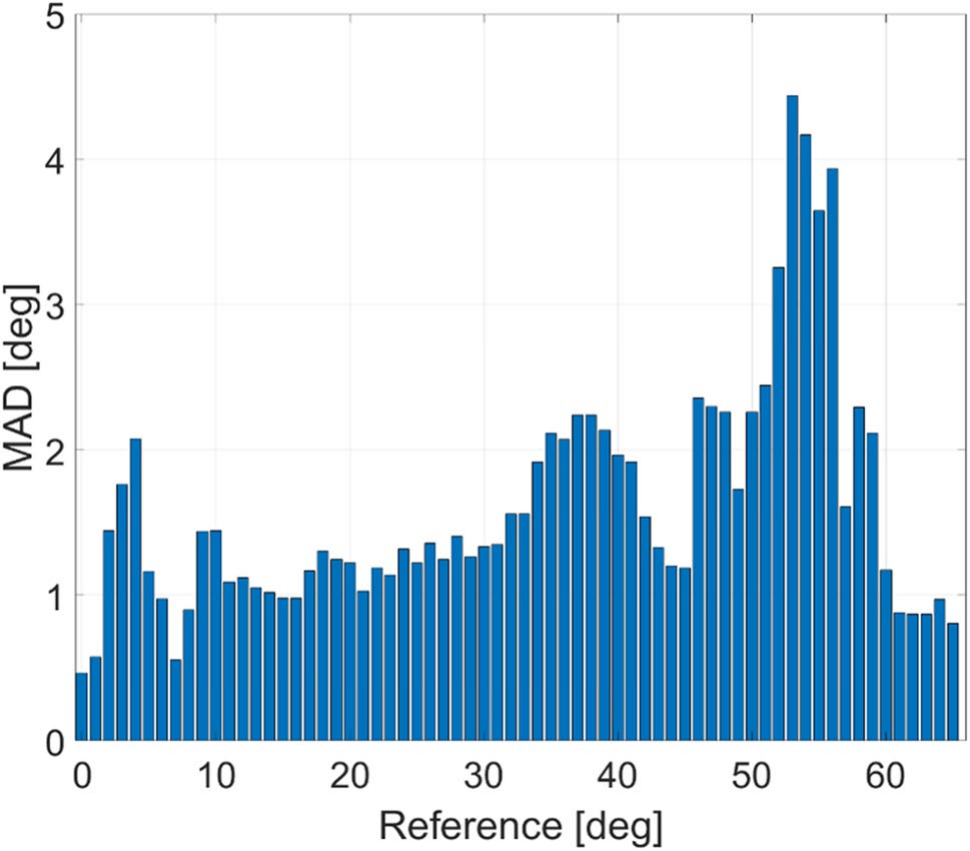
Histogram of the mean absolute difference (MAD) between *CA*_*per*_ and *Φ*_*per ref L5*_

Fifth, the consistency of the measured *CA*_*per*_ is assessed by comparing with the camera reference over a wide range of inflection angles occurring in the data measured from the subject. The difference between *CA*_*per*_ of the IMU data compared to Φ_*per ref L5*_ of the camera reference is calculated for each data sample and rounded to the nearest whole degree. The histogram of the mean absolute difference (MAD) of all angles is shown in Fig. 10. It shows a MAD limited to a range of approximately 2° for angles below 50°. Somewhat larger differences up to about 4.5° occur in the range between 50° and 56°. This can be explained by an increasing non-linearity of the IMU sensor data towards larger angles as well as limited validity of the anatomical simplifications made in (1) for estimating *CA*_*per*_ towards large bending angles.

## Discussion

The dummy test with a protractor confirms that manual measurement using the *NeuroPostureApp* and measurements from the motion capture system match well, indicating that the motion capture system is indeed a reliable reference for validating the system. It is important to note that the app and digital protractor are used in this work solely as a base for additional comparison and validation of the proposed IMU system. As such they are not part of the suggested final approach, consisting only of the IMU arrangement and the described algorithms. Therefore, this is not a limitation for replication or further developments, but it provides additional proof of the consistency and accuracy of our measurement system. The angles calculated from the IMU sensors showed a deviation on the order of +0.5°/−1.5°. These errors can be expected to contribute to the reported RMSE values. Additional error in the subject measurement may be attributed to skin movement in relation to the underlying bones (and hence sensor movement), particularly in the C7 region [34]. The proposed system was demonstrated with a 4-min recording of IMU and motion capture data obtained from a healthy volunteer performing various activities including a mimicked Parkinsonian gait. Longer recording periods up to a few hours are unproblematic and have been conducted in the authors’ lab. Automated recognition of the significant activities has been demonstrated in principle [35] and is a current subject to further investigation. The presented data show that the CA using the perpendicular method can successfully be obtained from two sensors. Assessing the leg angle with another two sensors and adding these angles implement the malleolus method. The RMSE values of *CA*_*per*_ fall within the range expected from a combination of IMU measurement error, motion artifacts, and model limitations. A small systematic error exists for the malleolus method since the reference point L5 moves relative to the hip joint during motion.

Validation of the leg angle *Φ*_*leg*_ was performed using *Φ*_*leg ref IC*_ obtained from visual markers on IC and LM, showing satisfactory accuracy. The RMSE values during still standing upright, still standing with forward bending, walking, and parkinsonian gait are 2.74°, 0.91°, 4.69°, and 2.94° respectively. Higher errors in *Φ*_*leg*_ are observed during the walking activity, which includes a free leg swinging phase that allows additional degrees of freedom of motion not included in the model. The use of only two sensors to measure the leg angle with high accuracy during walking is challenging. Gait is a unique characteristic of each individual and depends on various parameters, including step length and muscle force [12]. Han et. al [36] presented an algorithm for estimating the orientation angles of the thigh and shank using accelerometers and gyroscopes. Compared to a motion capture system, their algorithm exhibited RMSE of 2.9°, 3.6°, and 4.2° for flexion/extension (pitch), adduction/abduction (roll), and internal/external rotation (yaw) angles of the thigh and shank, respectively. Incorporating additional leg position information into the algorithm may further enhance the measurement of thigh (*φ*_*TH*_) and shank (*φ*_*SH*_*)* angles and ultimately improve leg angle estimation during walking. However, dynamic activities such as walking or parkinsonian gait are not a primary target activity for the PD studies intended here, and it is recommended to evaluate such phases separately aside the CA progression monitoring.

The RMSE of the measured *CA*_*per*_ and *CA*_*mal*_ in the standing still with forward bending activity were improved from 3.32° and 4.79° to 1.68° and 2.45°, respectively, after the patient specific calibration was performed in this position. It is the primary target activity for measuring CA. A different calibration position may be chosen for other target activities.

To put the observed error into further perspective, it is useful to compare with the results of other researchers using IMU for posture detection. A method for estimating the stooped posture using two acceleration sensors on the neck and upper back is described in [37]. The RMSE of estimated inclination angles for the neck and upper back were reported as 0.62° and 0.72°, respectively. However, that method only estimated angles for a small range of the spine using a single sensor in a specific location, whereas measuring the CA requires measuring the angle of the entire spine with cumulative higher error. A study presented in [34] utilized two IMUs to measure the 3D orientation of the trunk. It combined the accelerometer, gyroscope, and magnetometer outputs of the IMUs to estimate the trunk orientation of volunteers during both sports and anatomical trunk motions. RMSE values of 3.0° ± 1.3°, 4.6° ± 2.4°, and 3.0° ± 1.4° were obtained for the lateral flexion, axial rotation, and flexion/extension angles, respectively.

Table 4 compares the proposed approach with the established methods of still photo and camera-based measurement. Firstly, in terms of observation time, the proposed IMU system offers a long-term monitoring capability similar to camera systems, whereas photography provides only momentary data. Moreover, as far as its technical deployment is concerned, the proposed IMU system allows measurement at home, similar to photography, while camera systems typically require a clinical setting. As an out-of-clinic method, it carries typical associated disadvantages of operating in an uncontrolled environment, including data riddled with additional noise and interference, possible intermittent recording failure (e.g., when a sensor detaches), and post hoc misinterpretation of data. Since the proposed continuous monitoring method yields additional insights not available with conventional techniques, this is an acceptable trade-off in many applications. Activity detection is feasible with both the proposed IMU system and camera systems, whereas photography lacks this capability. The proposed IMU system, along with camera systems and photography, enables camptocormia angle detection. Additionally, both the proposed IMU system and traditional methods, including camera systems and photography, support the perpendicular and malleolus methods. Regarding accuracy, all methods exhibit high accuracy levels. However, the setup size and cost of the proposed IMU system are more moderate, compared to camera systems. Unlike camera systems and photography, the proposed IMU system has the potential to eliminate the white coat effect and enhance patient comfort.

**Table 4:** Comparison of the principle properties of different body position measurement approaches

	Camera system	Photograph	Proposed IMU system
Observation time	Long	Momentary	Long
Measurement at home	No	Yes	Yes
Activity detection	Yes	No	Yes
CA detection	Yes	Yes	Yes
Perp. and Malleolus methods	Yes	Yes	Yes
Accuracy	High	High	High
Setup size	Large	Small	Small
Setup cost	High	Low	Medium
White coat effect	Yes	Yes	No

It is worth noting that while the motion capture system used as a reference exhibits slightly higher accuracy compared to our proposed system, the additional advantages of our system especially for application outside the clinic position it as a viable enhancement. It may also find use in a clinical setting in cases where unobtrusive monitoring is paramount to a maximum accuracy obtained in the limited observation space of a camera system.

## Conclusion

The conventional methods of measuring CA using still photos or short video sequences can produce bias and, most importantly, are not suitable for continuous assessment of camptocormia over longer periods of time outside the laboratory environment. IMU sensors provide a suitably accurate and reliable way of measuring CA even during certain activities. An implementation of the malleolus measurement method (as an extension of the perpendicular method) is proposed and validated. It enables IMU assisted assessment of this consensus method, maintaining the reference to past studies. The proposed algorithm can, in principle, be easily implemented on a mobile device, e.g., a smartphone, to provide real-time monitoring of the CA. The measurements showed that the measured angle is stable over an expanded recording period, of which a 4-min window was evaluated here in more detail. Drift over time is neither observed nor expected since gravity is used as the absolute reference for the acceleration measurements and thus errors are not accumulated.

Data were obtained from a single, healthy volunteer of young age, who mimics parkinsonian gait. Therefore, a generalization of the findings to other age groups and patients with PD is limited. Investigations with actual Parkinson patients are currently under way in our laboratories and in the home environment, using the presented system as a valuable assessment tool.
